# Epidemiological Characteristics of Traumatic Spinal Cord Injury in Saudi Arabia: A Systematic Review

**DOI:** 10.7759/cureus.67531

**Published:** 2024-08-22

**Authors:** Albaraa M Almallah, Ghaida A Albattah, Asmaa A Altarqi, Amr A Al Sattouf, Khalid M Alameer, Dalal M Hamithi, Ryan D Alghamdi, Mohmmed S AlShammri, Bandar M Abuageelah, Abdulhadi Y Algahtani

**Affiliations:** 1 College of Medicine, Qassim University, Qassim, SAU; 2 Medicine and Surgery, Ibn Sina National College, Jeddah, SAU; 3 Faculty of Medicine, Jazan University, Jazan, SAU; 4 Faculty of Medicine, King Abdulaziz University, Jeddah, SAU; 5 College of Medicine, King Faisal University, Al Ahsa, SAU; 6 General Medicine Practice Program, Batterjee Medical College, Aseer, SAU; 7 Department of Neuroscience, King Abdulaziz Medical City, National Guard Health Affairs, Jeddah, SAU; 8 Research Office, King Abdullah International Medical Research Center, Jeddah, SAU; 9 College of Medicine, King Saud Bin Abdulaziz University, Jeddah, SAU

**Keywords:** injuries, trauma, spinal column, saudi arabia, epidemiology, spinal cord injuries

## Abstract

Traumatic spinal cord injury (TSCI) is a severe condition with high mortality and disability rates. Understanding the regional TSCI epidemiology may facilitate the development of targeted preventive initiatives and the optimization of resource allocation. The primary goal of this systematic review was to gather and analyze the existing literature on the frequency and characteristics of TSCI in Saudi Arabia. A literature search of PubMed, Web of Science, and Google Scholar was conducted in January 2024 following the Preferred Reporting Items for Systematic Reviews and Meta-Analyses guidelines. Observational studies reporting TSCI epidemiology in Saudi Arabia between 2010 and 2022 were included. Data on demographics, mechanisms, levels/severity, and outcomes were extracted. Methodological quality was assessed using the Newcastle-Ottawa Scale. Nine studies involving 2,356 TSCI cases were analyzed. Most patients were young males. Road traffic accidents were shown to be the predominant cause, accounting for 56.5-90.8% of cases. Thoracic (28.7-48.3%) and cervical (26.6-39%) levels were the most common. The extent of neurological deficits showed significant variation throughout the studies. This review provides a baseline understanding of TSCI epidemiology in Saudi Arabia but highlights critical gaps that future research should address. The review emphasizes the need for evidence-based interventions targeting road safety and falls, standardized cervical spine evaluation and management, and the use of validated metrics to optimize patient outcomes. Large-scale population-based studies with standardized methodologies are necessary to fully understand TSCI epidemiology, prognosis, and long-term disability burden in Saudi Arabia, leading to better prevention strategies and improved patient outcomes.

## Introduction and background

Spinal cord injury (SCI) is a severe condition that occurs when the spinal cord is injured from the base of the skull to the lower back, resulting in loss of sensation and motor abilities [1]. There are two classifications of SCIs, namely, traumatic injuries and nontraumatic injuries [2]. Traumatic spinal cord injury (TSCI) is considered one of the most severe and catastrophic types of injuries, leading to high mortality and disability rates. This condition causes physical and emotional challenges for patients, while also placing a considerable strain on families and society [3-5]. TSCI is the result of traumatic factors that cause injuries to neural structures within the spinal canal, including the spinal cord, nerve roots, and cauda equina [6].

The clinical outcomes of SCI depend on the severity and location of the lesion and may include partial or complete loss of sensory and/or motor function below the level of injury. SCI commonly results from a sudden, traumatic impact on the spine that fractures or dislocates vertebrae. The initial mechanical forces delivered to the spinal cord at the time of injury is known as primary injury where displaced bone fragments, disc materials, and/or ligaments bruise or tear into the spinal cord tissue. Regardless of the form of primary injury, these forces directly damage ascending and descending pathways in the spinal cord and disrupt blood vessels and cell membranes causing spinal shock, systemic hypotension, vasospasm, ischemia, ionic imbalance, and neurotransmitter accumulation [7].

The global incidence of TSCI has been estimated at 10.5 per 100,000 individuals [8]. Within Saudi Arabia, further research is needed to characterize epidemiological trends and severity levels associated with TSCI. Thus, this systematic review seeks to aggregate and analyze all available data related to the epidemiology of TSCI within Saudi Arabia. By synthesizing the existing literature, this study aims to enhance understanding of TSCI patterns and burdens affecting the Saudi population.

## Review

Methodology

Search Strategy

This systematic review was prospectively registered in PROSPERO (CRD42024508271) and conducted following the Preferred Reporting Items for Systematic Reviews and Meta-Analyses (PRISMA) guidelines. A literature search of PubMed, Web of Science, and Google Scholar was performed in January 2024 independently by two authors using the terms “spinal cord injury” OR “traumatic spinal cord injury” AND “Saudi Arabia” to identify epidemiological studies of TSCI in the country. Reference lists were also reviewed to find relevant articles. Studies reporting incidence, prevalence, causes, injury levels, age, and gender distributions were included.

Study Selection

**Figure 1:**
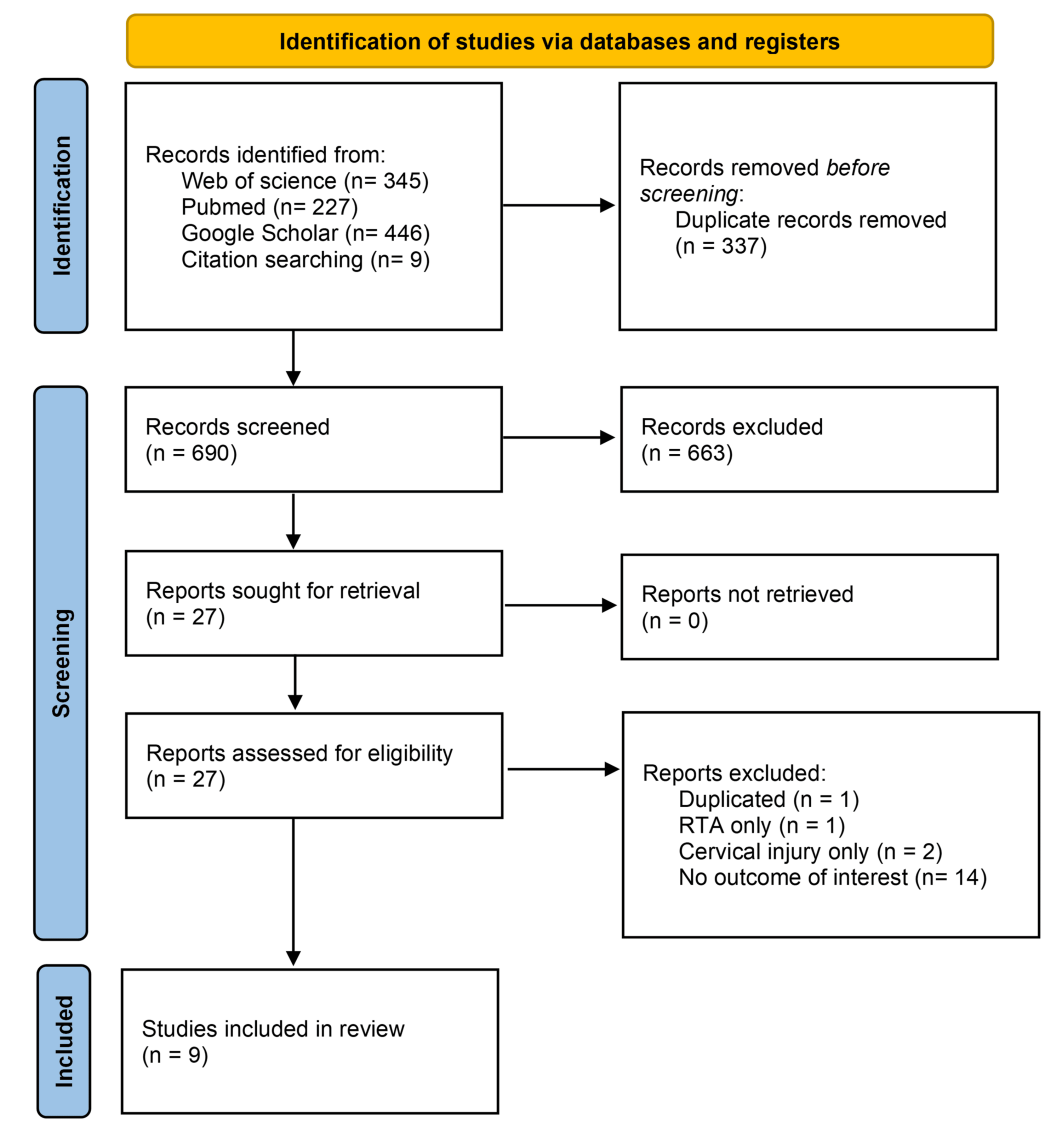
Preferred Reporting Items for Systematic Reviews and Meta-Analysis (PRISMA) flow diagram for study selection.

Five reviewers searched independently and screened titles and abstracts yielded by the search strategy to determine eligibility. Full potentially eligible texts were then independently assessed for inclusion against predefined criteria. Studies were included if they reported primary epidemiological data on TSCI in Saudi Arabia. Exclusion criteria involved nontraumatic etiologies, nonoriginal study designs, lack of outcomes of interest, and non-Saudi population applicability. Discrepancies were resolved through consensus discussion. Adherence to the PRISMA flow diagram (Figure 1) provided transparency in the systematic selection process.

Analyzed Variables

Data extraction focused on key epidemiological variables using a standardized form. Study characteristics included duration, location, design, and setting. Demographic data comprised case numbers, mean/median age, age groups, and gender distribution. Injury details involved mechanisms, spinal level, and severity. Outcomes such as length of stay, complications, and mortality were also extracted. Where possible, incidence/prevalence rates were calculated. Geographic information and time trend analyses were noted when available. Correspondence with authors addressed unclear data. This systematic approach facilitated a comprehensive understanding of TSCI characteristics in Saudi Arabia.

Quality Assessment

Three reviewers independently assessed methodological quality using the Newcastle-Ottawa Scale (NOS) for observational studies. The NOS evaluates the selection and comparability of groups and the outcome assessment on a nine-point scale. Studies scoring 6 points or above, representing higher quality based on the instrument’s selection criteria, comparability, and outcome identification, were considered to be of superior methodological rigor.

Results

The database search yielded nine studies (Figure 1) that met the study’s selection criteria involving 2,356 cases with TSCI in Saudi Arabia. These studies were primarily retrospective, published between 2010 and 2022, and conducted at tertiary centers. Most studies reported the etiology, level, and severity of injury, along with other epidemiological parameters. Sample sizes ranged widely, from just 23 patients in the smallest study to 495 cases in the largest.

Demographic Characteristics

Consistently, in all the studies analyzed, the vast majority of patients were male, with proportions varying from 74% to 90%. The study by Al-Habib et al. [12] found the lowest ratio of males to females, with a ratio of 2.8:1. Conversely, the study by Mehdar et al. [15] reported the highest ratio, with a ratio of 23:3. Among the studies analyzed in this review, one specifically focused on children under 18 years of age, with a mean age of 13.7 years. Seven articles documented the mean ages of patients with TSCI across all age groups, which ranged from 29 to 35 years. Table 1 provides further information regarding the demographic characteristics of the patients.

**Table 1:** A summary of the published data concerning patients with TSCIs. TSCI = traumatic spinal cord injury; M = male; F = female

Study ID (reference)	Duration of the study	Study design	Region/City	Source population	Total cases	Mean age (SD)	Male (%)	M/F ratio
Al-Jadid and Robert 2010 [9]	January 2005 to October 2008	Retrospective	Riyadh	Sultan Bin Abdulaziz Humanitarian City (SBAHC)	495	34.3 ± 0.68	404 (81.6%)	4.43:1
Alshahri et al. 2012 [10]	January 2003 to December 2008	Retrospective	Riyadh	Riyadh Military Hospital (RMH)	307	29.5	271 (88%)	7.5:1
Al-Jadid 2013 [11]	August 1982 to November 2010	Retrospective	Riyadh	Prince Sultan Military Medical City (PSMMC)	466	29.7 ± 0.73	398 (85.4%)	6:1
Al-Habib et al. 2014 [12]	May 2001 to May 2009	Retrospective	Riyadh	King Abdulaziz Medical City (KAMC)	23	13.7	17 (74%)	2.8:1
Alshahri et al. 2016 [13]	January 2012 to December 2015	Retrospective	Riyadh	King Fahad Medical City (KFMC)	216	28.94	187 (86.6%)	6.4:1
Mahmoud et al. 2017 [14]	2009 to December 2014	Retrospective cohort study	Riyadh	King Fahad Medical City (KFMC)	312	35 ± 17	244 (78%)	3.5:1
Mehdar et al. 2019 [15]	June 2018 to June 2019	Retrospective	Najran	King Khalid hospital (KKH)	182	-	161 (88.4%)	23:3
Alnaami et al. 2021 [16]	January 2016 to December 2018	Retrospective search of electronic records	Abha	Aseer Central hospital (ACH)	112	32.1 ± 14.12	101 (90.2%)	9:1
Dibas et al. 2022 [17]	January 2017 to December 2019	Retrospective	Qassim	Buraidah Central Hospital (BCH)	243	35	194 (79.8%)	4:1

Etiology and Level of TSCIs

Among the nine studies included in the analysis, seven provided details regarding the causes or origins of the injury. Road traffic accidents were consistently identified as the main factor in all studies, accounting for 56.5% to 90.8% of cases. Falls were the second most common cause of TSCIs, representing 3.2% to 35% of all injuries. A study by Mehdar et al. revealed that falls were a prevalent factor contributing to TSCI in males and females, accounting for 15% and 7% of cases, respectively [15]. According to another study, falls accounted for 40% of TSCIs in females [12]. In a study conducted on children, falls were identified as the second most prevalent cause of TSCI in patients who were under the age of 12 years [12]. Falls accounted for 42.8% of injuries in individuals aged 56 years and older [13]. Less common causes of injury included sports-related incidents, occupational mishaps, acts of violence, and gunshot wounds. The level of SCI was documented in four studies [11,12,16,17]. The cervical and thoracic spine had the highest prevalence in TSCI cases, ranging from 26.6% to 39% and 28.7% to 48.3%, respectively. Table 2 provides detailed information on the etiology and levels of TSCIs.

**Table 2:** Etiology and level of injury in the included studies. RTA = road traffic accident

Study ID (reference)	Etiology				Level of injury			
	RTA	Falls	Penetrating wound	Others	Cervical	Thoracic	Lumbar	Sacral
Al-Jadid and Robert 2010 [9]	-	-	-	-	-	-	-	-
Alshahri et al. 2012 [10]	262 (85%)	28 (9%)	Gunshot: 14 (5%)	Diving: 3 (1%)	-	-	-	-
Al-Jadid 2013 [11]	377 (80.1%)	44 (9.4%)	Gunshot: 30 (6.5%) Stab knife: 5 (1%)	Diving: 3 (0.6%) Object falling: 7 (1.5%)	146 (31.3%)	225 (48.3%)	95 (20.4%)	0
Al-Habib et al. 2014 [12]	13 (56.5%)	8 (35%)	0	2 (8.6%)	9 (39%)	11 (47.8%)	Lumbosacral: 10 (43.5%)	
Alshahri et al. 2016 [13]	196 (90.8%)	7 (3.2%)	13 (6%)	0	-	-	-	-
Mahmoud et al. 2017 [14]	-	-	-	-	-	-	-	-
Mehdar et al. 2019 [15]	116 (64%)	40 (22%)	Gunshot: 15 (8%)	Bomb blast: 7 (4%)	-	-	-	-
Alnaami et al. 2021 [16]	89 (79.50%)	23 (20.50%)	0	0	40 (35.7%)	37 (33%)	35 (31.3%)	0
Dibas et al. 2022 [17]	162 (66.7%)	78 (32.1%)	0	3 (1.23%)	63 (26.6%)	68 (28.7%)	133 (56.1%)	5 (2.11%)

Severity of Injury and Mortality

To comprehend prognostic variables and healing patterns after TSCI, outcome assessment is crucial. Only a small percentage of the studies included in this systematic review used standardized assessment measures to report injury severity and functional outcomes. Only three studies reported the severity of injury based on the American Spinal Injury Association (ASIA) impairment scale [14,16,17]. The ASIA is a standardized neurological examination used to assess SCI-related sensory and motor impairment. The scale has five levels, ranging from complete neural function loss to normal. Grade A SCIs eliminate all motor and sensory functions, including sacral segments, distal to the injury site. However, incomplete injuries (B to D) retain motor or sensory function below the injury site. Grade B injuries have sensory but no motor function, while Grade C injuries have motor grades less than three below the neurologic level. Motor function is indicated by a motor grade of at least three below the neurologic level in grade D injuries. Grade E injuries show normal motor and sensory examinations but may have abnormal reflexes or neurologic phenomena. The study by Mahmoud et al. [14] reported that 84% of cases were classified as ASIA type A. In the study by Dibas et al., ASIA type E was the most common, accounting for 82.6% of cases, while in the study by Alnaami et al., it was 43.8%. Mortality was mentioned in two studies and ranged between 2% and 2.5% [15,17]. Further details on the severity of injury and mortality can be found in Table 3.

**Table 3:** Severity of injury and mortality in the included studies. ASIA = American Spinal Injury Association

Study ID (reference)	Severity of the injury									
	ASIA A	ASIA B	ASIA C	ASIA D	ASIA E	Complete paraplegia	Complete tetraplegia	Incomplete paraplegia	Incomplete tetraplegia	Mortality
Al-Jadid and Robert 2010 [9]	-	-	-	-	-	-	-	-	-	-
Alshahri et al. 2012 [10]	-	-	-	-	-	90 (29%)	66 (21%)	56 (18%)	95 (31%)	-
Al-Jadid 2013 [11]	-	-	-	-	-	-	-	-	-	-
Al-Habib et al. 2014 [12]	-	-	-	-	-	-	-	-	-	0
Alshahri et al. 2016 [13]	-	-	-	-	-	80 (37%)	36 (16.7%)	53 (24.5%)	47 (21.7%)	-
Mahmoud et al. 2017 [14]	261 (84%)	25 (8%)	26 (8%)	0	-	239 (77%)	73 (23%)	-	-	-
Mehdar et al. 2019 [15]	-	-	-	-	-	9 (5%)	5 (3%)	-	-	4 (2%)
Alnaami et al. 2021 [16]	34 (30.4%)	6 (5.4%)	16 (14.3%)	7 (6.3%)	49 (43.8%)	-	-	-	-	-
Dibas et al. 2022 [17]	8 (3.40%)	12 (5.11%)	7 (2.98%)	14 (5.96%)	194 (82.6%)	9 (3.8%)	6 (2.5%)	-	-	6 (2.5%)

Evaluation of Methodological Quality

The NOS rated all studies as good or very good, signifying a low risk of bias. Seven investigations achieved scores of at least 3 to 5, while two attained a score of 6. As shown in Table 4, methodological quality was sound overall, with isolated minor concerns for some. This quality evaluation implies that results were likely dependable despite occasional shortcomings.

**Table 4:** Quality assessment table of the included studies using the Newcastle-Ottawa Scale for assessing the methodological quality of observational studies.

Study ID (reference)	Selection	Comparability	Outcome	Overall quality
Al-Jadid and Robert 2010 [9]	*	*	*	Good
Alshahri et al. 2012 [10]	**	*	**	Good
Al-Jadid 2013 [11]	**	*	**	Good
Al-Habib et al. 2014 [12]	**	*	**	Good
Alshahri et al. 2016 [13]	**	*	**	Good
Mahmoud et al. 2017 [14]	**	**	**	Very good
Mehdar et al. 2019 [15]	**	*	**	Good
Alnaami et al. 2021 [16]	**	*	**	Good
Dibas et al. 2022 [17]	**	**	**	Very good

Discussion

This systematic review provides a comprehensive overview of the country’s current understanding of TSCI epidemiology, drawing insights from a robust analysis of nine observational studies published between 2010 and 2022. The findings underscore the substantial burden of TSCI, with 2,356 cases reported across the included investigations. When considering these findings alongside previous research, certain consistencies and differences emerge. A 2023 meta-analysis from China estimated the global incidence of TSCI at 65.15 per million (95% confidence interval (CI) = 47.20-83.10/million), with a range of 6.7 to 569.7 per million, primarily due to motor vehicle accidents and falls. The most common types of TSCI were incomplete quadriplegia and AISA/Frankel grade D [18]. A 2009-2020 review of developing nations reported an incidence of 22.55 per million annually (95% CI = 13.52; 37.62/million/year), predominantly affecting males [19]. A 2018 Middle East-North Africa review found an incidence of 23.24 per million annually (95% CI = 5.64-49.21), primarily impacting young males via motor vehicle accidents, followed by falls. Complete paraplegia was the most common type of injury, with thoracic-level injuries being the most prevalent [20]. Our analysis corroborates motor vehicle accidents and falls as the chief causes in Saudi Arabia. It also aligns with international evidence implicating young males as the highest risk. Given the country’s historical ban on women driving until 2018, this is not surprising, given the prevalence of young males involved in automobile collisions as drivers or passengers. However, injury characteristics reported locally vary from region to region.

A prospective observational study at Prince Mohammed bin Abdulaziz Hospital from January 2017 to June 2018 aimed to determine the prevalence of spinal trauma in adults in Saudi Arabia. Out of 230 patients, 90.0% were male, with 60% in their second and third decades. Motor vehicle accidents caused 83% of cases, with 50% being drivers and 80% being unrestrained passengers. About 50% of spinal injuries were linked to injuries in other body parts. Cervical spine injuries made up 44% of cases, followed by lumbar spine injuries [21]. We conducted a thorough analysis of nine observational studies, which included a total of almost 2,356 cases, to get a comprehensive grasp of this topic. To improve understanding, it is imperative to address certain notable limitations identified in all of the research studies presented. Automobile accidents were shown to be the primary cause of SCIs in the majority of cases, as indicated by the research. Women in Saudi Arabia were not allowed to drive until 2018 due to the country’s laws. Thus, there were many accounts of automobile collisions involving young males who were either driving or riding as passengers in the age range of 20 to 29. This indicates that implementing injury prevention strategies specifically aimed at this high-risk demographic, such as more stringent enforcement of traffic safety legislation and public awareness campaigns, might potentially reduce the incidence of serious SCIs caused by car accidents [22].

Multiple research projects have shown that cervical spine fractures and dislocations are the most often-seen types of anatomical injuries. This indicates that in high-energy trauma scenarios such as vehicle accidents, the neck is particularly vulnerable [23]. Enhanced automobile and road designs that focus more on cervical spine protection might help prevent some of these accidents. Hospitals throughout the country need to establish and implement early care protocols and procedures particularly designed for cervical spine trauma. However, there was significant variation seen in the severity and scope of neurological abnormalities documented in the studies [24,25]. The lack of consistent assessment and reporting of outcomes at different follow-up periods made it difficult to make accurate comparisons. To accurately diagnose injuries and assess the progress of healing, it is necessary to use standardized classification and assessment methods that rely on trustworthy neurological tests, such as the American Spinal Injury Association Impairment Scale [26-28]. Saudi Arabia presently does not have extensive multicenter research that utilizes standardized procedures.

Moreover, because exposures and outcomes have already occurred, the retrospective observational designs of all studies included in the analysis may be prone to confounding biases [29,30]. Using medical records to gather injury facts instead of obtaining prospective data increases the possibility of memory bias. The results might be influenced by selection bias if certain patient subgroups were excluded. Prospective cohort studies that follow patients from the time of injury until rehabilitation may provide data of superior quality. The sample sizes exhibited significant variation; studies related to a specific facility had fewer than 100 occurrences [31]. The limited sample size is a challenge to conducting rigorous statistical analyses for risk factor modeling and subgroup evaluations. It is not feasible to accurately determine regional differences in the causes and outcomes of medical conditions throughout Saudi Arabia’s many geographic areas based only on a case series from one institution [32,33]. It is necessary to conduct larger multi-site cohorts that include thousands of cases throughout the nation to examine any variations at the population level.

The length of the follow-up varied and often just included in-hospital therapy, with no information provided on the long-term functional status. The interpretation of recovery trajectories and rehabilitation needs is severely restricted. Similarly, it was unable to determine the trends in healthcare use and the costs associated with the long-term management of chronic illnesses [34,35]. It is essential to conduct studies that include long-term follow-ups of patients in the community. Loss to follow-up was often recorded but inadequately quantified, perhaps resulting in attrition bias. The grounds for not disclosing the subsequent losses. Some patients may have had challenges in their ongoing rehabilitation, experienced difficulties, or, unfortunately, succumbed, all of which might have an indeterminate effect on long-term outcomes [36,37]. Enhancing validity would be achieved by reducing attrition via dedicated monitoring efforts.

It is necessary to accurately describe the epidemiology of TSCI in Saudi Arabia, even though this review provides some useful first results. Prospective multicenter cohort studies with standardized methodologies are crucial for enrolling a large number of patients throughout the country [38-42]. To address the current gaps, it is necessary to actively collect extensive data on injury exposure, employ standardized categorization systems, analyze patterns of healthcare consumption, measure outcomes over a long period, conduct analyses on risk factors, minimize biases caused by attrition, and investigate regional differences. Prioritizing the enhancement of preventative, acute care, rehabilitation, and community reintegration services might be achieved by implementing a coordinated national registry or monitoring system, which would also maximize future research efforts. Nationally coordinated prospective research using standardized procedures is required to fully describe the injury spectrum, examine trends over time, assess outcomes, and guide evidence-based preventive and care measures specific to the Saudi population.

## Conclusions

A systematic review study has not compiled epidemiological information on TSCI in Saudi Arabia. This review synthesized available epidemiological data on TSCI in Saudi Arabia between 2010 and 2022. Significant results showed that road accidents result in a high burden of illness, particularly impacting young male patients who frequently experience thoracic and cervical injuries, leading to a considerable strain on the healthcare system. Nevertheless, firm conclusions are impossible due to the methodological flaws of observational research. Our study highlights the need for evidence-based interventions to address the leading causes of TSCI in Saudi Arabia. Targeted road safety and fall prevention campaigns may help address the leading causes implicated. Clinical practices should prioritize standardized cervical spine evaluation and management protocols, while rehabilitation services should incorporate validated metrics such as ASIA to optimize patient outcomes. Future research should focus on large-scale, population-based studies employing standardized methodologies to comprehensively understand TSCI epidemiology, prognostic trajectories, and long-term disability burdens. By refining our knowledge, we can inform targeted prevention strategies and enhance clinical management and rehabilitation planning, ultimately improving patient outcomes and reducing the burden of TSCI in Saudi Arabia.
